# Biologically Active Transition Metal Chelates of Ni(II), Cu(II) and Zn(II) With 2-Aminothiazole-Derived Schiff-bases: Their Synthesis, Characterization and the Role of Anions (NO_3_, SO_4_^2-^, C_2_O_4_^2-^and CH_3_CO_2-_) on Their Antibacterial Properties

**DOI:** 10.1155/MBD.1999.187

**Published:** 1999

**Authors:** Zahid H. Chohan

**Affiliations:** Department of Chemistry Islamia University Bahawalpur Pakistan

## Abstract

Biologically active nickel(ll), copper(ll) and zinc(ll) chelates with thiazole-derived nitro- and chlorosalicylaldehyde Schiff-bases having the same metal ion but different anions, e.g. nitrate, sulfate, oxalate and acetate have been synthesized and characterized on the basis of their physical, spectral and analytical data. In order to evaluate the possible participating role of anions on the antibacterial properties, these ligands and their synthesized metal chelates with various anions have been screened against bacterial species *Escherichia coli*, *Pseudomonas aeruginosa* and *Staphylococcus aureus*.


### BIOLOGICALLY ACTIVE TRANSITION METAL CHELATES OF Ni(II), Cu(II) AND Zn(II) WITH 2-AMINOTHIAZOLE-DERIVED SCHIFF-BASES: THEIR SYNTHESIS, CHARACTERIZATION AND THE ROLE OF ANIONS ($NO_3$, $SO_4^{2-}$, $C_2O_4^{2-}$ AND $CH_3CO_2^-$) ON THEIR ANTIBACTERIAL PROPERTIES

Zahid H. Chohan

Department of Chemistry, Islamia University, Bahawalpur, Pakistan

### ABSTRACT

Biologically active nickel(II), copper(II) and zinc(II) chelates with thiazole-derived nitro- and chlorosalicylaldehyde Schiff-bases having the same metal ion but different anions, e.g. nitrate, sulfate, oxalate and acetate have been synthesized and characterized on the basis of their physical, spectral and analytical data. In order to evaluate the possible participating role of anions on the antibacterial properties, these ligands and their synthesized metal chelates with various anions have been screened against bacterial species *Escherichia coli, Pseudomonas aeruginosa* and *Staphylococcus aureus.*

### INTRODUCTION

Various thiazole compounds have been found to be associated with diverse antibacterial[1], antifungal[2], antitumour[3], anticancer[4] and anthelmintic[5] activities. Many studies[6-10] have described the biological role of metals and chelation and have established the fact that those anticancer drugs as viable ligands exhibit increased anticancer activity when administered as their metal chelates. The connected metal centers in such biologically active molecules may involve different functions such as oxygen transport, DNA inhibitor, enzymatic activity, electron transfer and lock geometry. All these observations related to the essential role of metals linkage attracted our attention to commence a systematic research program[11-15] to investigate the factors responsible for this relationship. During the course of previous investigations thiazole-derived nitro- and chlorosalicylaldehyde Schiff-bases and their various transition metal chelates were prepared[16] as antibacterial agent and studied for this relationship of metal chelation and the subsequent antibacterial properties. In continuation to the same, during the present work we wish to extend the same studies hitherto the less investigated possible participating biological role of anions on these prepared chelates, which stay as the counterpart of the complexes. For this purpose thiazole-derived nitro- and chlorosalicylaldehyde Schiff-bases ($HL^1$ and $HL^2$) (Fig. 1) and their metal chelates of the type $[M(L)_2X]$ [M=Ni(II), Cu(II) and Zn(II), L=$HL^1$ & $HL^2$ (Fig.1) and X= $NO_3$, $SO_4^{2-}$, $C_2O_4^{2-}$ and $CH_3CO_2^-$] having the same metal ion but different anions were synthesized, characterized and screened for their antibacterial activity against *Escherichia coli, Staphylococcus aureous* and *Pseudomonas aeruginosa*

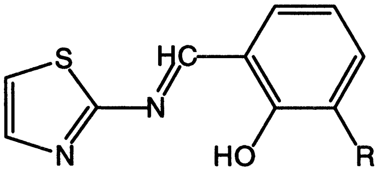

$HL^1$: R = $NO_2$; $HL^2$: R = Cl

**Figure 1.** Structure of the Schiff-bases

### EXPERIMENTAL

### Material and Methods

All chemicals and solvents used were of Analar grade. All metal were used as their nitrates, sulfates, oxalates and acetates. IR spectra were recorded on a Philips Analytical PU 9800 FTIR Spectrophotometer. UV-Visible spectra were obtained on a Hitachi U-2000 double-beam spectrophotometer. Conductance of the metal complexes was determined in DMF on a YSI-32 model conductometer. Magnetic measurements were done on solid complexes using the Gouy method. Melting points were recorded on a Gallenkamp apparatus and are uncorrected. The antibacterial studies were carried out with the help of the Department of Pathology, Quaid-e-Azam Medical College, Bahawalpur, Pakistan.

**Preparation of Schiff-bases**

The ligands were prepared by adopting the same procedure as reported earlier[16].

**Preparation of Metal Complexes**

An ethanol solution of appropriate metal(II) salt (1 mmol, 20 mL) was added to a stirred hot ethanol solution of the respective Schiff-base (2 mmol, 30 mL). The resulting mixture was refluxed for 3 h. The solution was then cooled, filtered, reduced to nearly half its volume and then left for two days at room temperature. During this period the product crystallized. It was filtered, washed with ethanol and ether and dried to give the desired metal complexes **1** (62 %), **2** (60 %), **3** (58 %), **4** (61 %), **5** (60 %), **6** (62 %), **7** (59 %), **8** (60 %), **9** (62 %), **10** (58 %), **11** (61 %), **12** (62 %), **13** (61), **14** (60 %), **15** (59 %), **16** (58 %), **17** (62 %), **18** (60 %), **19** (62 %), **20** (59 %), **21** (60 %), **22** (61 %), **23** (63 %) and **24** (60 %).

**Antibacterial Studies**

The synthesized metal chelates in comparison to the free ligand were screened for their antibacterial activity against bacterial species *Escherichia coli, Staphylococcus aureus* and *Pseudomonas aeruginosa*. The paper disc diffusion method was used for the determination of the antibacterial activity.

**Preparation of Discs.**

The ligand/complex (30 μg) in DMF (0.01mL) was applied on a paper disc, [prepared from blotting paper (3 mm diameter)] with the help of a micropipette. The discs were left in an incubator for 48 h at 37° C and then applied on the bacteria grown agar plates.

**Preparation of Agar Plates.**

Minimal agar was used for the growth of specific bacterial species. For the preparation of agar plates for *Escherichia coli*, MacConkey agar (50 g), obtained from Merck Chemical Company, was suspended in freshly distilled water (1 L). It was allowed to soak for 15 minutes and then boiled on a water bath until the agar was completely dissolved. The mixture was autoclaved for 15 minutes at 120° C and then poured into previously washed and sterilized Petri dishes and stored at 40° C for inoculation.

**Procedure of Inoculation.**

Inoculation was done with the help of a platinum wire loop which was made red hot in a flame, cooled and then use for the application of bacterial strains.

**Application of Discs.**

A sterilized forceps was used for the application of paper disc on the already inoculated agar plates. When the discs were applied, they were incubated at 37° C for 24 h. The zone of inhibition was then measured (in diameter) around the disc.

### RESULT AND DISCUSSIONS

**Physical Properties**

Schiff-bases (Fig. 1) were prepared by reacting equimolar amounts of nitro- or chlorosalicylaldehyde and 2-aminothiazole respectively in ethanol. The crystallized products after characterization were reported earlier[16] and now used further for the preparation of its Ni(II), Cu(II) and Zn(II) complexes having different anions as $NO_3$, $SO_4^{2-}$, $C_2O_4^{2-}$ and $CH_3CO_2^-$.

All the prepared complexes are crystalline solids and melt with decomposition above 245° C without showing sharp melting points. The complexes are soluble in DMF, DMSO and benzene. Their melting behavior, solubility and crystalline nature suggested that they are non-polymeric. Elemental analysis data (Table 1) indicated 1:2 (metal:ligand) stoichiometry. Low conductance values (11-18 $ohm^{-1}$ $cm^2$ $mol^{-1}$) indicated that the complexes are non-electrolytes[17,18].

**Infrared Spectra**

The detailed IR spectra of the ligands[16] show a weak band at ~ 2900 $cm^{-1}$ instead of a broad medium band at 3100 $cm^{-1}$. This might be due to intramolecular hydrogen bonding between the phenolic (–OH) hydrogen and the nitrogen of the azomethine group[19]. The absence of νOH in the complexes suggested the deprotonation of the phenolic –OH group of the Schiff-base and its co-ordination through oxygen atom. Moreover, the νC-O and νC=N modes which appeared at ~ 1280 and ~ 1630 $cm^{-1}$ respectively in the Schiff-bases were shifted in the complexes. This shifting of νC-O towards higher frequency (~ 1330-1345 $cm^{-1}$) and lowering of νC=N (~ 1590-1620 $cm^{-1}$) suggested that the co-ordination of the Schiff-bases occurred through the deprotonated oxygen of the phenolic –OH group and nitrogen of the azomethine (HC=N) groups[19-21].

Further conclusive evidence of the co-ordination of the ligand with the metal was confirmed by the appearance of weak low frequency bands at ~ 485-520 and ~ 355-370 $cm^{-1}$ (Table 2) due to metal-oxygen and metal-nitrogen stretching vibrations[22] in the metal complexes and not observed in the spectra of Schiff-bases.

**Table 1.** Physical and Analytical Data of Metal(II) Chelates

| No | Metal chelate/Mol. Formula | M.P($^oC$) (decom) | B.M. ($\mu_{eff}$) | Cal (Found)% C H N |
|---|---|---|---|---|
| 1 | $[Ni(L_1)_2(NO_3)_2]$ $C_{20}H_{12}NiN_8O_{12}S_2$ [678.82] | 252-254 | 3.10 | 35.4 1.8 16.5 (35.7)(2.0)(16.2) |
| 2 | $[Ni(L_1)_2(SO_4)]$ $C_{20}H_{12}NiN_6O_{10}S_3$ [650.88] | 264-266 | 2.91 | 36.9 1.8 12.9 (37.2)(2.2)(12.5) |
| 3 | $[Ni(L_1)_2(C_2O_4)]$ $C_{22}H_{12}NiN_6O_{10}S_2$ [642.82] | 258-260 | 2.96 | 41.1 1.9 13.1 (41.5)(2.1)(12.8) |
| 4 | $[Ni(L_1)_2(CH_3CO_2)_2]$ $C_{24}H_{18}NiN_6O_{10}S_2$ [672.82] | 255-257 | 3.20 | 42.8 2.7 12.5 (42.9)(2.5)(12.3) |
| 5 | $[Ni(L_2)_2(NO_3)_2]$ $C_{20}H_{12}NiClN_6O_8S_2$ [622.27] | 250-252 | 3.10 | 38.6 1.9 13.5 (38.9)(2.0)(13.1) |
| 6 | $[Ni(L_2)_2(SO_4)]$ $C_{20}H_{12}NiClN_4O_6S_3$ [594.33] | 261-262 | 2.90 | 40.4 2.0 9.4 (40.9)(1.8)(9.9) |
| 7 | $[Ni(L_2)_2(C_2O_4)]$ $C_{22}H_{12}NiClN_4O_6S_2$ [586.27] | 258-260 | 3.10 | 45.0 2.0 9.6 (45.6)(2.4)(9.2) |
| 8 | $[Ni(L_2)_2(CH_3CO_2)_2]$ $C_{24}H_{18}NiClN_4O_6S_2$ [616.27] | 252-254 | 2.98 | 46.7 2.9 9.1 (46.8)(3.2)(8.8) |
| 9 | $[Cu(L_1)_2(NO_3)_2]$ $C_{20}H_{12}CuN_8O_{12}S_2$ [683.66] | 255-257 | 1.92 | 35.1 1.8 16.4 (35.4)(2.2)(16.1) |
| 10 | $[Cu(L_1)_2(SO_4)]$ $C_{20}H_{12}CuN_6O_{10}S_3$ [655.72] | 248-250 | 1.87 | 36.6 1.8 12.8 (36.9)(2.0)(12.5) |
| 11 | $[Cu(L_1)_2(C_2O_4)]$ $C_{22}H_{12}CuN_6O_{10}S_2$ [647.66] | 250-252 | 1.91 | 40.8 1.9 13.0 (41.0)(2.2)(13.5) |
| 12 | $[Cu(L_1)_2(CH_3CO_2)_2]$ $C_{24}H_{18}CuN_6O_{10}S_2$ [677.66] | 245-247 | 1.90 | 42.5 2.7 12.4 (42.1)(2.9)(12.0) |
| 13 | $[Cu(L_2)_2(NO_3)_2]$ $C_{20}H_{12}CuClN_6O_8S_2$ [627.11] | 255-257 | 1.88 | 38.3 1.9 13.4 (38.7)(2.4)(13.3) |
| 14 | $[Cu(L_2)_2(SO_4)]$ $C_{20}H_{12}CuClN_4O_6S_3$ [599.17] | 254-256 | 1.89 | 40.1 2.0 9.3 (40.3)(2.4)(9.1) |
| 15 | $[Cu(L_2)_2(C_2O_4)]$ $C_{22}H_{12}CuClN_4O_6S_2$ [591.11] | 252-254 | 1.91 | 44.7 2.0 9.5 (44.9)(2.2)(9.7) |
| 16 | $[Cu(L_2)_2(CH_3CO_2)_2]$ $C_{24}H_{18}CuClN_4O_6S_2$ [621.11] | 250-252 | 1.90 | 46.4 2.9 9.0 (46.5)(3.3)(9.1) |
| 17 | $[Zn(L_1)_2(NO_3)_2]$ $C_{20}H_{12}ZnN_8O_{12}S_2$ [685.5] | 258-260 | Dia | 35.0 1.8 16.3 (35.2)(2.1)(16.5) |
| 18 | $[Zn(L_1)_2(SO_4)]$ $C_{20}H_{12}ZnN_6O_{10}S_3$ [657.56] | 264-266 | Dia | 36.5 1.8 12.8 (36.3)(2.0)(12.6) |
| 19 | $[Zn(L_1)_2(C_2O_4)]$ $C_{22}H_{12}ZnN_6O_{10}S_2$ [649.5] | 261-263 | Dia | 40.6 1.8 12.9 (40.9)(2.1)(13.3) |
| 20 | $[Zn(L_1)_2(CH_3CO_2)_2]$ $C_{24}H_{18}ZnN_6O_{10}S_2$ [679.50] | 260-262 | Dia | 42.4 2.6 12.4 (42.1)(2.8)(12.3) |
| 21 | $[Zn(L_2)_2(NO_3)_2]$ [628.95] | 263-265 | Dia | 38.2 1.9 13.4 (38.7)(2.2)(13.7) |
| 22 | $[Zn(L_2)_2(SO_4)]$ $C_{20}H_{12}ZnClN_4O_6S_3$ [601.01] | 265-267 | Dia | 40.0 2.0 9.3 (40.4)(2.2)(9.1) |
| 23 | $[Zn(L_2)_2(C_2O_4)]$ $C_{22}H_{12}ZnClN_4O_6S_2$ [592.95] | 259-261 | Dia | 44.5 2.0 9.4 (44.7)(2.6)(9.5) |
| 24 | $[Zn(L_2)_2(CH_3CO_2)_2]$ $C_{24}H_{18}ZnClN_4O_6S_2$ [622.95] | 260-262 | Dia | 46.2 2.9 9.0 (46.5)(3.1)(8.8) |

### UV-Visible spectra and Magnetic moments

The electronic spectra of the Ni(II) complexes exhibited four bands at 7915-8955, 14225-14875, 24225-24760 and 29550-30125 $cm^{-1}$. The first three bands were assigned to the spin-allowed transitions $^3A_2g(v_1) \rightarrow ^3T_2g$, $^3A_2g(v_2) \rightarrow ^3T_1g(F)$ and $^3A_2g(v_3) \rightarrow ^3T_1g(P)$ respectively. The fourth band at 29550-30125 $cm^{-1}$ was of high intensity band due to ligand-metal charge-transfer. The occurrence of three spin-allowed transitions supported an octahedral geometry for the Ni(II) complexes[23]. The magnetic moments of Ni(II) complexes also lie in the range 2.9-3.2 B.M (Table 1) and confirm the octahedral geometry[24].

**Table 2.** Spectral Data of Metal(II) Chelates

| No | IR ($cm^{-1}$) | $\lambda_{max}$ ($cm^{-1}$) |
|---|---|---|
| 1 | 1330 (C-O), 1620 (HC=N), 480 (M-O), 345 (M-N) | 29550, 24760, 14545, 8955 |
| 2 | 1345 (C-O), 1615 (HC=N), 485 (M-O), 355 (M-N) | 29775, 24225, 14875, 8270 |
| 3 | 1135 (C-O), 1595 (HC=N), 490 (M-O), 350 (M-N) | 30125, 24790, 14285, 8555 |
| 4 | 1345 (C-O), 1610 (HC=N), 495 (M-O), 355 (M-N) | 29555, 24265, 14570, 7915 |
| 5 | 1330 (C-O), 1602 (HC=N), 505 (M-O), 365 (M-N) | 30225, 24775, 14545, 8345 |
| 6 | 1335 (C-O), 1590 (HC=N), 510 (M-O), 355 (M-N) | 29750, 24315, 14555, 8280 |
| 7 | 1335 (C-O), 1595 (HC=N), 505 (M-O), 365 (M-N) | 30510, 24790, 14225, 8550 |
| 8 | 1340 (C-O), 1620 (HC=N), 495 (M-O), 360 (M-N) | 29655, 24280, 14570, 7975 |
| 9 | 1340 (C-O), 1615 (HC=N), 490 (M-O), 345 (M-N) | 27110, 14235 |
| 10 | 1338 (C-O), 1610 (HC=N), 495 (M-O), 345 (M-N) | 27245, 14530 |
| 11 | 1330 (C-O), 1605 (HC=N), 485 (M-O), 355 (M-N) | 28500, 15350 |
| 12 | 1345 (C-O), 1620 (HC=N), 510 (M-O), 365 (M-N) | 28170, 15100 |
| 13 | 1330 (C-O), 1620 (HC=N), 480 (M-O), 345 (M-N) | 29245, 14530 |
| 14 | 1345 (C-O), 1615 (HC=N), 485 (M-O), 355 (M-N) | 29375, 14530 |
| 15 | 1135 (C-O), 1595 (HC=N), 490 (M-O), 350 (M-N) | 28450, 14875 |
| 16 | 1345 (C-O), 1610 (HC=N), 495 (M-O), 355 (M-N) | 28170, 15345 |
| 17 | 1330 (C-O), 1602 (HC=N), 505 (M-O), 365 (M-N) | 28250 |
| 18 | 1335 (C-O), 1590 (HC=N), 510 (M-O), 355 (M-N) | 29255 |
| 19 | 1335 (C-O), 1595 (HC=N), 505 (M-O), 365 (M-N) | 29515 |
| 20 | 1340 (C-O), 1620 (HC=N), 495 (M-O), 360 (M-N) | 28275 |
| 21 | 1340 (C-O), 1615 (HC=N), 490 (M-O), 345 (M-N) | 29250 |
| 22 | 1338 (C-O), 1610 (HC=N), 495 (M-O), 345 (M-N) | 28445 |
| 23 | 1330 (C-O), 1605 (HC=N), 485 (M-O), 355 (M-N) | 28270 |
| 24 | 1345 (C-O), 1620 (HC=N), 510 (M-O), 365 (M-N) | 29355 |

The electronic spectra of the Cu(II) complexes exhibited two bands, a broad unsymmetrical band in the visible region at 14235-15350 $cm^{-1}$ may be due to $^2E_g \rightarrow ^2T_{2g}$ transitions for their octahedral geometry and a sharp band of high intensity at 27110-29375 $cm^{-1}$ assigned to ligand-metal charge-transfer[25]. The magnetic moments of the Cu(II) complexes lie in the range 1.87-1.92 B.M expected[26,27] for their distorted octahedral environment. The electronic spectra of the Zn(II) complexes exhibited only a high intensity band at 28250-29515 $cm^{-1}$ assigned[28] to ligand-metal charge-transfer.

In the light of the above discussion an octahedral structure for the Ni(II) and Zn(II) chelates and a distorted octahedral structure for the Cu(II) chelates is proposed and it is tentatively proposed that the Schiff-bases coordinate through the nitrogen of the azomethine group, nitrogen of thiazole ring and deprotonated oxygen of phenolic group forming a stable chelate ring (Fig 2).

### Antibacterial Studies

The antibacterial activity of Schiff-bases in comparison to its metal chelates having the same metal atom but different anions was studied against bacterial species *Escherichia coli*, *Staphylococcus aureus* and *Pseudomonas aeruginosa*. Paper disc diffusion method reported[29-31] earlier was adopted for screening. The Schiff-bases and their complexes individually exhibited varying degrees of inhibitory effects on the growth of the tested bacterial species. The antibacterial results reproduced in Table 3 evidently show that the activity of the Schiff-bases became more

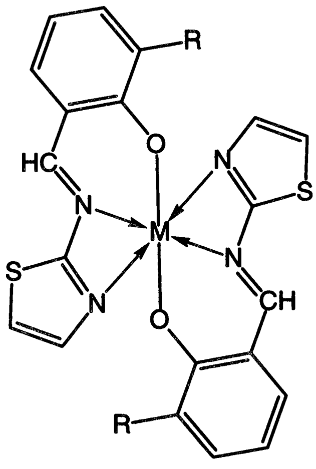

M=Ni(II), Cu(II) or Zn(II)

**Fig. 2.** Proposed Structure of the Metal(II) chelate

**Table 3** Antibacterial Activity Data

| Schiff-base/ Chelate | Microbial Species a | b | c |
|---|---|---|---|
| $HL^1$ | + | ++ | + |
| $HL^2$ | ++ | + | + |
| 1 | +++ | +++ | +++ |
| 2 | +++ | +++ | +++ |
| 3 | +++ | ++++ | +++ |
| 4 | +++ | ++ | ++ |
| 5 | ++++ | +++ | +++ |
| 6 | +++ | +++ | +++ |
| 7 | ++++ | +++ | +++ |
| 8 | ++ | +++ | + |
| 9 | +++ | +++ | ++++ |
| 10 | +++ | +++ | +++ |
| 11 | +++ | ++++ | ++ |
| 12 | ++ | ++++ | +++ |
| 13 | +++ | +++ | +++ |
| 14 | +++ | +++ | +++ |
| 15 | +++ | ++++ | +++ |
| 16 | +++ | ++ | ++ |
| 17 | ++++ | +++ | +++ |
| 18 | +++ | +++ | +++ |
| 19 | ++++ | +++ | +++ |
| 20 | ++ | +++ | + |
| 21 | +++ | +++ | ++++ |
| 22 | +++ | +++ | +++ |
| 23 | +++ | ++++ | ++ |
| 24 | ++ | ++++ | +++ |

a= *Escherichia coli*, b= *Staphylococcus aureus*, c= *Pseudomonas aeruginosa*
Inhibition zone diameter mm (% inhibition): +, 6-10 (27-45 %); ++, 10-14 (45-64 %); +++, 14-18 (64-82 %); ++++, 18-22 (82-100 %). Percent inhibition values are relative to inhibition zone (22 mm) of the most active compound with 100 % inhibition.

pronounced when co-ordinated to these metals. When the same metal chelate having different anions was individually screened the degree of bactericidal activity/potency also varied. From the obtained data, it was generally observed that the order of potency in comparison to the metal complexes having chloride anions evaluated and reported earlier[16] and to the results of the present studies against the same tested bacterial species under the same conditions were found to follow the order as:

$$NO_3 > C_2O_4 > CH_3CO_2 > Cl > SO_4$$

On the basis of these results, it is strongly claimed that different anions dominantly effect the biological behavior of the metal chelates. It is however suspected that factors such as solubility, conductivity, dipole moment and cell permeability mechanisms are certainly influenced by the presence of these anions in the chelate and may cause in increasing this activity/potency. Further *in vivo* studies are in progress, which would help the authors to establish the real mechanism and role of anions in increasing this biological activity.

### ACKNOWLEDGEMENT

The author gratefully acknowledges the help of Department of Pathology, Qaid-e-Azam Medical College, Bahawalpur, in undertaking the antibacterial studies.

### REFERENCES

1. B. Webster,. *Chem. Abstr.*, 1969, **71**: 1240.
2. S. C. Sharma,. *Bull. Chem. Soc. Jpn.*, 1967, **40**: 2422.
3. B. Dash and M. K. Raut,. *J. Ind. Chem. Soc.*, 1955, **32**: 663.
4. D Modi, S. S Sabnis and C. V Deliwala, *J. Med. Chem.*, 1970, **13**: 935.
5. D. K. Johnson, T. B. Murphy, T. B. Rose, W. H. Goodwin and L Pickart, *Inorg. Chim. Acta.*, 1982, **67**: 159.
6. D. R. Williams, "*The Metals of Life*", Van Nostrand, London, 1971.
7. D. R. William, *Chem. Rev.*, 1972, **72**: 203.
8. A. J. Crowe, "*Metal Based Antitumour Drugs*", Vol 1, M. Gielen (Ed), Freund, London, 1988.
9. A. Albert, *Aust. J. Sc.*, 1967, **30**: 1.
10. A. J. Crim and H. G. Petering, *Cancer. Res.*, 1967, **27**: 1109.
11. Z. H. Chohan and H. Pervez, *Synth. React. Inorg. Met.-Org. Chem.*, 1993, **23**: 1061.
12. Z. H Chohan and A. Rauf, *Synth. React. Inorg. Met.-Org. Chem.*, 1996, **26**: 591.
13. Z. H. Chohan, S. K. A. Sherazi and M. S. Iqbal, *Metal-Based Drugs.*, 1998, **5**: 347.
14. Z. H Chohan and S. K. A. Sherazi, *Synth. React. Inorg. Met.-Org. Chem.*, 1999, **29**, 105.
15. Z. H. Chohan, M. Praveen and S. K. A. Sherazi, *Metal-Based Drugs.*, 1998, **5**: 267.
16. Z. H. Chohan, *Metal-Based Drugs*, (In Press).
17. A. M. Shallary, M. M. Moustafa and M. M. Bekheit, *J. Inorg. Nucl. Chem.*, 1979, **41**: 267.
18. W. J. Geary, *Coord. Chem. Rev.*, 1971, **7**: 81.
19. J. E. Kovacic, *Spectrochim. Acta.*, 1967, **23A**: 183.
20. J. F. Jackowitz, J. A. Durkin and J. G. Walter, *Spectrochim. Acta.*, 1967, **23A**: 67.
21. D. M. Adams, "*Metal Ligand and Related Vibrations*", Edward Arnold, London, 1967.
22. K. Nakamoto, "*Infrared Spectra of Inorganic and Coordination Compounds*", John Wiley, New York, 1963.
23. A. B. P. Lever, "*Inorganic Electronic Spectroscopy*", 2nd Ed, Elsevier, New. York, 1984.
24. R. S. Drago, "*Physical Methods in Inorganic Chemistry*", Reinhold, New. York, 1965.
25. B. N. Figgis, "*Introduction to Ligand Fields*", Interscience, New. York, 1966.
26. L. M. Ciampolini and V. Campigili, *Inorg. Chim.*, 1965, **4**: 407.
27. D. W. Meek, R. S. Drago and T. S. Piper, *Inorg. Chem.*, 1962, **1**: 285.
28. C. J. Balhausen, "*Introduction to Ligand Field*", Mc Graw Hill, New York, 1962.
29. Z. H. Chohan, and A. Rauf, *J. Inorg. Biochem.*, 1992, **46**: 41.
30. Z. H. Chohan and S. Kausar, *Chem. Pharm. Bull.*, 1992, **40**: 2555.
31. Z. H. Chohan, *Chem. Pharm. Bull.*, 1992, **39**: 1578.

**Received: June 4, 1999 - Accepted: June 25, 1999 -**
**Received in revised camera-ready format: June 28, 1999**

